# Nationwide consensus on the clinical management of treatment-resistant depression in Italy: a Delphi panel

**DOI:** 10.1186/s12991-023-00478-7

**Published:** 2023-11-23

**Authors:** Giuseppe Maina, Marina Adami, Giuseppe Ascione, Emi Bondi, Domenico De Berardis, Dario Delmonte, Silvia Maffezzoli, Giovanni Martinotti, Alessandra Nivoli, Elena Ottavianelli, the Delphi Panel Collaboration Group, Tiziano Acciavatti, Umberto Albert, Sara Andreoli, Ileana Andriola, Fausto Antonielli Romanini, Roberta Bassetti, Francesca Bettini, Graziella Boi, Paolo Cacciani, Paola Calò, Alessandro Carano, Ilaria Casolaro, Stefania Chiappini, Paola Clemente, Virginia D’Ambrosio, Giacomo d’Andrea, Tiziana Dario, Pasquale De Fazio, Renato de Filippis, Francesco Di Carlo, Marco Di Nicola, Luca Di Paolo, Giampaolo Di Piazza, Gabriele Di Salvo, Monica Fiori, Alessandro Gentile, Matteo Lupi, Mirko Manchia, Matteo Marcatili, Livio Marchiaro, Vassilis Martiadis, Giulia Menculini, Giovanni Migliarese, Gaetano Nappi, Domenica Nucifora, Miriam Olivola, Claudia Palumbo, Elena Paschetta, Ettore Pasculli, Enrico Pessina, Federica Pinna, Marianna Pinto, Davide Piu, Donato Gerolamo Posadinu, Fabiola Raffone, Valerio Ricci, Ilario Ritacco, Gianluca Rosso, Elisa Simonini, Antonio Ventriglio, Andrea Fagiolini

**Affiliations:** 1 Department of Neuroscience “Rita Levi Montalcini”, University of Turin, Turin, Italy; 2 Psychiatric Unit, San Luigi Gonzaga University Hospital, Orbassano, Turin, Italy; 3 Department of Medical Affairs - Neuroscience, Janssen-Cilag SpA, Cologno Monzese, Italy; 4 Department of Mental Health and Addictions, ASST Papa Giovanni XXIII , Bergamo, Italy; 5 Department of Mental Health, ASL 4, Teramo, Italy; 6 Department of Neuroscience, Imaging, Clinical Sciences University G.d’Annunzio, Chieti-Pescara, Italy; 7 Department of Medicine, Surgery and Pharmacy, University of Sassari, Medical School, AOU-Sassari, Sassari, Italy; 8 FULLCRO Srl, Via Ignazio Guidi 3, 00147 Rome, Italy; 9 Department of Molecular Medicine, University of Siena, Siena, Italy; 10 Pescara, Italy; 11 Trieste, Italy; 12 Aprilia (LT), Italy; 13 Bari, Italy; 14 Padova, Italy; 15 Milano, Italy; 16 Rovato (BS), Italy; 17 Cagliari, Italy; 18 Brescia, Italy; 19 Lecce, Italy; 20 Ascoli Piceno, Italy; 21 Roma, Italy; 22 Torino, Italy; 23 Chieti, Italy; 24 Brindisi, Italy; 25 Catanzaro, Italy; 26 Pisa, Italy; 27 Arezzo, Italy; 28 Sassari, Italy; 29 Termoli (CB), Italy; 30 Monza, Italy; 31 Cuneo, Italy; 32 Napoli, Italy; 33 Perugia, Italy; 34 Pavia, Italy; 35 Messina-Taormina (ME), Italy; 36 Saluzzo e Savigliano (CN), Italy; 37 Bra (CN), Italy; 38 Lodi, Italy; 39 La Spezia, Italy; 40 Foggia, Italy

**Keywords:** Treatment-resistant depression, Major depressive disorder, Antidepressants, Consensus statement, Customized treatment, Esketamine

## Abstract

**Background:**

Treatment-resistant depression (TRD) is defined by the European Medicines Agency as a lack of clinically meaningful improvement after treatment, with at least two different antidepressants. Individual, familiar, and socio-economic burden of TRD is huge. Given the lack of clear guidelines, the large variability of TRD approaches across different countries and the availability of new medications to meet the need of effective and rapid acting therapeutic strategies, it is important to understand the consensus regarding the clinical characteristics and treatment pathways of patients with TRD in Italian routine clinical practice, particularly in view of the recent availability of esketamine nasal spray.

**Methods:**

A Delphi questionnaire with 17 statements (with a 7 points Likert scale for agreement) was administered via a customized web-based platform to Italian psychiatrists with at least 5 years of experience and specific expertise in the field of depression. In the second-round physicians were asked to answer the same statements considering the interquartile range of each question as an index of their colleagues’ responses. Stata 16.1 software was used for the analyses.

**Results:**

Sixty panellists, representative of the Italian territory, answered the questionnaire at the first round. For 8/17 statements more than 75% of panellists reached agreement and a high consensus as they assigned similar scores; for 4 statements the panellists assigned similar scores but in the middle of the Likert scale showing a moderate agreement with the statement, while for 5 statements there was indecision in the agreement and low consensus with the statement.

**Conclusions:**

This Delphi Panel showed that there is a wide heterogeneity in Italy in the management of TRD patients, and a compelling need of standardised strategies and treatments specifically approved for TRD. A high level of consensus and agreement was obtained about the importance of adding lithium and/or antipsychotics as augmentation therapies and in the meantime about the need for long-term maintenance therapy. A high level of consensus and agreement was equally reached for the identification of esketamine nasal spray as the best option for TRD patients and for the possibility to administrate without difficulties esketamine in a community outpatient setting, highlighting the benefit of an appropriate educational support for patients.

**Supplementary Information:**

The online version contains supplementary material available at 10.1186/s12991-023-00478-7.

## Background

The primary goal of treating depression is to achieve complete resolution of symptoms, but approximately 30% of patients with major depressive disorder (MDD) do not respond adequately to treatment [1, 2]. Non-response to medication is common and can persist after multiple attempts with different medications [2]. The success rate of treatment decreases with each subsequent trial, as shown in the Sequenced Treatment Alternatives to Relieve Depression (STAR-D) trial [1]. Treatment-resistant depression (TRD) is defined by the European Medicines Agency (EMA) as a lack of clinically meaningful improvement after treatment with at least two different antidepressants [3]. TRD is a complex condition influenced by genetic, clinical and environmental factors, as well as comorbidities and psychosocial factors [4]. Patients with TRD experience a higher burden of illness compared to responders, including more severe symptoms, greater disability, and reduced quality of life [2, 5]. The economic burden of TRD is also significant, with higher direct and indirect costs compared to non-treatment-resistant depression [6]. Current management of TRD is challenging due to the lack of evidence-based guidelines or a consensus strategy in Europe, leading to variation in treatment choices [7]. Pharmacological options, that include selective serotonin reuptake inhibitors (SSRI), serotonin–norepinephrine reuptake inhibitors (SNRI), tricyclic antidepressants (TCA), monoamine oxidase inhibitors (MAOIs), and atypical antidepressants and non-pharmacological treatments (neurostimulation, psychotherapeutic interventions) could be used, alone or in combination, with different strategies, such as dose escalation, medication switching, combination therapy, and augmentation/additional therapy. The everyday Italian clinical practice is not different from the European context; in Italy it can be documented, on the one hand the frequent use of SSRI, SNRI and augmentation strategies, and on the other hand the rare utilization of psychosocial approaches [5].

However, in real-world practice, treatment response rates are low. A recent observational study on TRD in Europe confirmed that TRD patients have a poor chance of achieving remission at both 6 and 12 months; moreover, the study found that patients who had achieved remission at 6 months were then unable to maintain it for a long time [7]. Despite low remission rate, TRD patients often remain on the same pharmacological treatment for extended periods of time [7, 8]. There is a need for additional therapeutic strategies for TRD that are rapid acting and have proven efficacy in this population [4, 9, 10]. Ketamine and its S-enantiomer, esketamine, have shown promise in targeting the glutamate pathway and restoring synaptic connections in the brain to improve mood symptoms [11, 12]. Esketamine nasal spray, developed and approved specifically for TRD, provides an additional treatment option with rapid onset of action and demonstrated efficacy compared with other well-established pharmacological strategy such as augmentation with quetiapine XR [13]. Few adverse events are reported with esketamine (the most common are transient dissociative symptoms, nausea, dizziness) [14] and the safety concerns can be managed by administering esketamine under healthcare professional supervision in accordance with best practices [15]. Cost-utility analysis suggests that esketamine may be a cost-effective option for the treatment of TRD [16]. Future developments in pharmacological treatments of TRD are testing ketamine derivatives or other glutamatergic agents. In addition, GABAergic agents (e.g., zuranolone), opioid receptor and voltage- gated ion channels modulators, orexin antagonists, but also anti-inflammatory, as well as thyroid hormones are under investigation in TRD [15].

Given the lack of clear guidelines and the availability of new medications, it is important to understand the consensus regarding the clinical characteristics and treatment pathways for patients with MDD and TRD in routine clinical practice, particularly regarding esketamine nasal spray.

## Methods

The Delphi technique, developed in 1962 [17], derives the name from the Delphic oracle’s skills of interpretation and foresight; it is a process used to achieve a consensus concerning real-world knowledge from experts about certain areas. Delphi is a well-established methodology used in the scientific field [18, 19]. The Delphi process traditionally begins with a small group of experts preparing a questionnaire based upon an extensive review of the literature; this questionnaire is used as the instrument of the survey. Each Delphi participant is asked to review and rate the summarized statements so that areas of consensus and non-consensus can be identified. Each Delphi participant receives, in subsequent rounds, a questionnaire that includes the statements and ratings (from the previous round) and are asked to re-evaluate their initial judgment.

### Expert board and consensus panel

In September 2022, a board of 6 experts, based on their documented expertise in the TRD field, met to review the current landscape of the disease and identify key topics for clinical management. All members of the expert board disclosed potential conflicts of interest.

At the end of the topic selection process, replies and redundancies were eliminated and 17 statements were generated for testing across a wider audience using the Delphi questionnaire.

The statements can be grouped as follows:

- Clinical characteristics and diagnosis of patients with TRD (statements: 1, 2, 3)
- Treatment journey and organizational implications (statements: 4, 5, 6, 13, 16, 17)
- Antidepressant treatment in routine clinical practice (statements 7, 8, 9, 10, 11, 12, 14, 15).

The panellists have been identified by the experts board according to the following criteria, decided during the first meeting and were asked for volunteer participation:

- specialized in psychiatry, with specific expertise in the field of depression (at least 100 patients/year) and direct or indirect experience with esketamine nasal spray;
- years of experience (at least 5 including specialization);
- working in the Italian National Health Service (public service, outpatient/territorial setting, University in agreement with NHS);
- representative of the Italian territory.

### Questionnaire and statistical analyses

The Delphi questionnaire was administered via a web-based system. The platform used for the data collection, called "NPCdata_survey DE9 Version 1.0" is dedicated to the management of Delphi conferences. The system has been validated according to GAMP V guidelines and resides in a protected area on ARUBA servers. Data integrity security is guaranteed by ARUBA back-up systems and Fullcro's internal procedures. The access to the system was done through LogIn. Each user was assigned a unique code and link to the system.

This method granted anonymity and absence of interference among the panellists. The link to the web was sent by e-mail with a maximum of one reminder.

The definitions for consensus and non-consensus were decided a priori. A Likert scale was used (1 = no agreement to 7 = maximum agreement) to evaluate the degree of agreement with each of the statements proposed in the questionnaire.

In the first round, the user logged into the system and provided a score to all the statements (mandatory responses). After saving, the access is removed to prevent any change to the answers provided.

At the end of the first round (October 27–November 15, 2022), the median value and the 25th and 75th percentiles (75th p–25th p, interquartile range) of each statement were calculated.

In the second round (21 November–19 December 2022), the system presented, for each statement, the answers provided by the user in the first round and the interquartile ranges calculated across the entire database (which represents the range in which 50% of the answers fell) as an index of their colleagues’ responses. Those who answered outside the interquartile range (IQR) in the second round were asked by the system to give a reason for their response. In the absence of this information the system does not save the session. At the end of the second round, the median value and the 25th and 75th percentiles of each statement and IQR were calculated again.

The results of the first and second rounds and the motivations of those who had answered outside the interquartile range, were discussed by the expert board at the “verification meeting” (January 2023). After discussing and commenting on the results of each of the 17 statements, the expert board members formulated the counter-motivations. Since all 17 statements reached agreement and consensus; it was decided not to proceed with the third round.

**Fig. 1:**
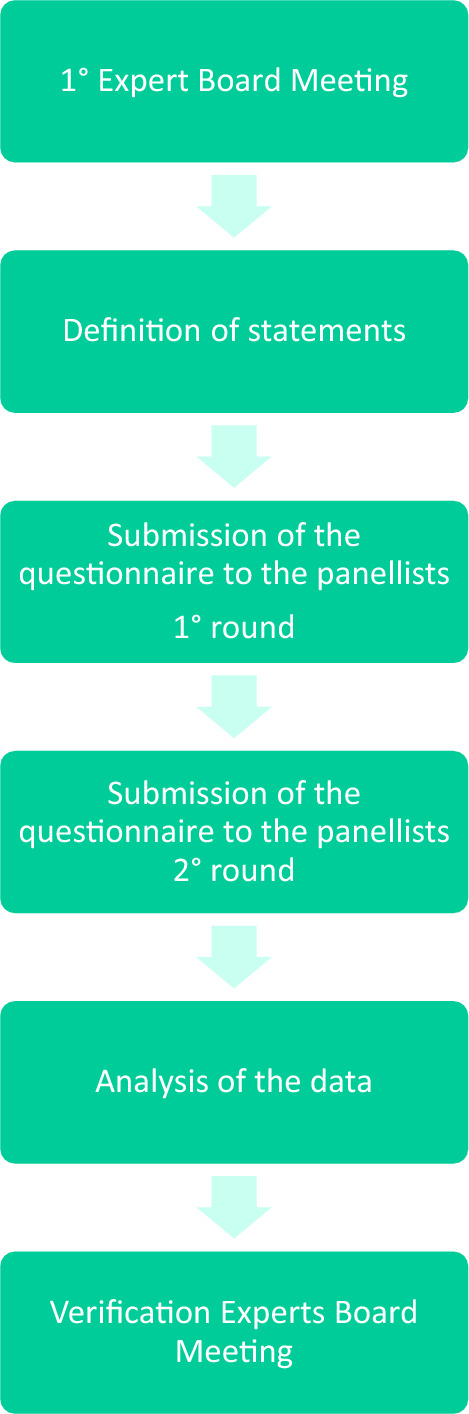
Flow chart of the Delphi process. The Delphi process begins with the experts board preparing a list of statements based upon an extensive review of the literature. Each Delphi participant is asked to answer the statements according to a Likert scale from 1 to 7. Each Delphi participant receives, in a second round, a questionnaire that includes the same statements and the interquartile range of each question (which represents the range in which 50% of the answers fell) as an index of their colleagues’ responses (from the previous round) and are asked to re-evaluate their initial judgment. Those who answered outside the interquartile range (IQR) in the second round were asked to give a reason for their response. At the end of the second round, the median value and the 25th and 75th percentiles of each statement and IQR were calculated again. The results of the first and second rounds and the motivations of those who had answered outside the interquartile range, were discussed by the expert board at the “verification meeting”

The flow chart of the analysis is presented in Fig. 1.

**Fig. 2:**
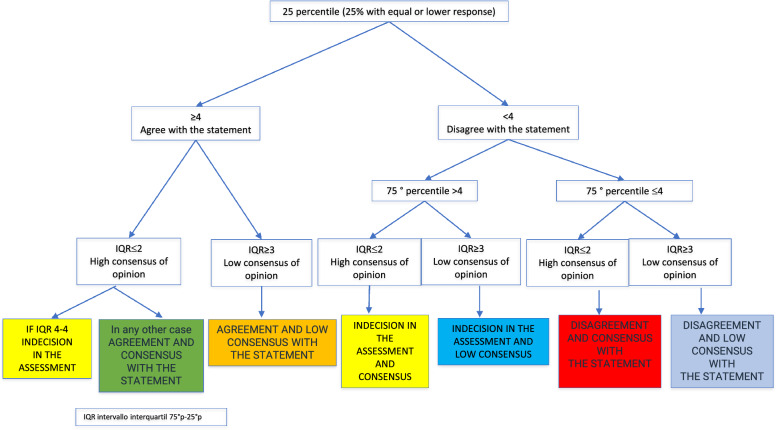
Ranking of the statements. The statements were ranked based on the 25th percentile, 75th percentile and the interquartile range (IQR). The results were classified as in agreement, indecision or in disagreement with the statement and on the other hand, reaching consensus or low consensus. The combination of these two indicators creates different categories: agreement and consensus, agreement and low consensus, indecision in the assessment and consensus, indecision in the assessment and low consensus, disagreement and consensus, disagreement and low consensus

The statements were ranked based on the 25th percentile, 75th percentile and the interquartile range (IQR) (Fig. 2):

- Agreement and consensus with the statement: Affirmations that have the 25th percentile ≥ 4 and IQR ≤ 2 but different from 4 to 4 belong to this group.
- Agreement and low consensus with the statement: Those statements that have the 25th percentile ≥ 4 and IQR ≥ 3 belong to this group.
- Indecision in the evaluation and consensus with the statement: Those statements that have the 25th percentile ≥ 4 and IQR 4–4 or 25th percentile < 4 and 75th percentile > 4 and IQR ≤ 2 belong to this group.
- Indecision in the evaluation and low consensus with the statement: Those statements that have the 25th percentile < 4, 75th percentile > 4 and IQR ≥ 3.
- Disagree and consensus with the statement: Those statements that have the 25th percentile < 4, 75th percentile ≤ 4 and IQR ≤ 2 belong to this group.
- Disagree and low consensus with the statement: Those statements that have the 25th percentile < 4, 75th percentile ≤ 4 and IQR ≥ 3 belong to this group.

Median and 25th percentile, 75th percentile and IQR were reported for each statement and round (Additional file 1).

Stata 16.1 software was used for the analyses.

## Results

### Participants

Sixty panellists answered the questionnaire at the first round, and 58 at the second round.

With regard to the geographic distribution, the panel was well representative of the Italian situation (33% North, 20% Centre, 47% South and Islands). Among the 60 participants 21 worked in universities, 39 in public structures as Department of Mental Health, organized in day-care Mental Health Centers or Hospital Diagnosis and Treatment of Psychiatric Services.

**Fig. 3:**
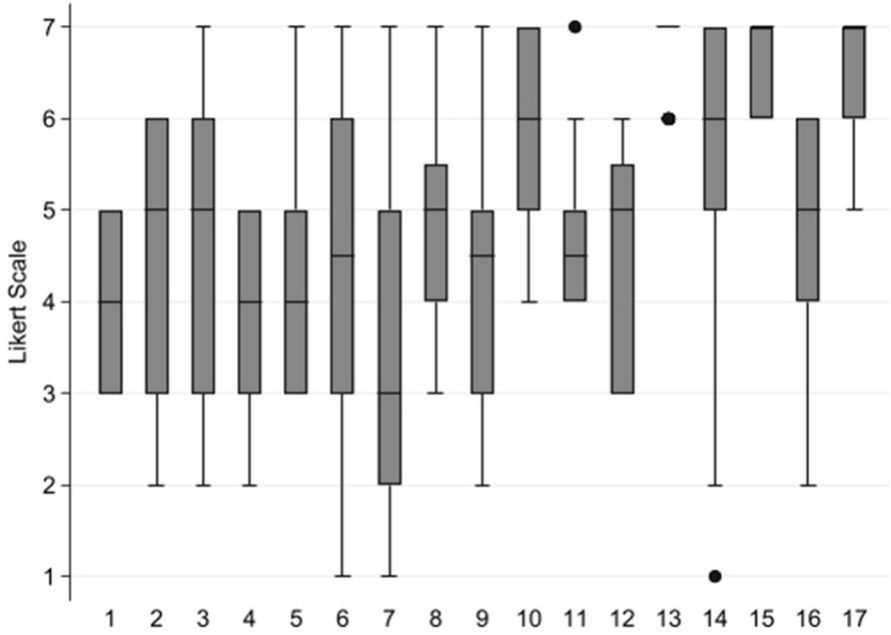
Level of agreement/disagreement. Results distribution of each statement at Round 2

The overall result of the 17 statements is summarized in Table 1 and displayed in graphical form (level of agreement/disagreement distribution by box plot and bar graph) in Fig. 3.

**Table 1:** Summarization of the results of the 17 statements

		Consent	Agreement
1.	In my opinion environmental and personological factors are among the main factors responsible for the non-response to treatment of almost all patients with TRD	+	±
2.	In my clinical practice I also consider as treatment resistant a patient with incomplete improvement of symptoms (partial response) after an adequate period of treatment	−	±
3.	In clinical practice I usually use scales/questionnaires for the diagnostic classification and/or evaluation of the clinical course of the patient with depression	−	±
4.	In my opinion a shared strategy is being pursued in Italy (based on guidelines, evidence-based treatments, diagnostic-therapeutic pathways) for the management of patients with TRD	+	±
5.	Based on my clinical experience, I believe that after the second treatment failure there is a clear reduction in remission chances	+	±
6.	When I treat a depressed patient, if after 3–4 weeks there is no response, I decide to change the antidepressant therapy	−	±
7.	When treating a depressed patient who has failed to respond to two antidepressants, I believe the best option is to combine an antipsychotic, such as quetiapine	−	±
8.	When treating a depressed patient who has failed to respond to two antidepressants, I do tend to add lithium first	+	+
9.	In treating a depressed patient who has failed to respond to two antidepressants I do tend to associate psychotherapy first	+	±
10.	When treating a depressed patient who has failed to respond to two antidepressants, I believe the best option is to combine esketamine (if currently available or when available in my centre)	+	+
11.	I am satisfied with the efficacy of lithium and/or antipsychotics as augmentation therapies for patients with TRD	+	+
12.	I use (or I will use) esketamine nasal spray only after non-response to the available augmentation/increase strategies	−	±
13.	After obtaining a satisfactory response to treatment in a patient with TRD, long-term maintenance of therapy is essential	+	+
14.	In my opinion most patients with TRD can be treated with esketamine nasal spray in a community outpatient setting, without difficulties	+	+
15.	Educational support for patients helps to make the best use of the therapeutic opportunity offered by esketamine nasal spray	+	+
16.	In my daily reality, I have adequate and sufficient resources (staff, logistics, facilities, etc.) to provide patients with TRD with the best possible care	+	+
17.	In my opinion there are aspects of professional responsibility that the clinician must take into consideration in order to prefer, when possible, drugs with approved clinical indications for patients with TRD	+	+

For 8 among the 17 statements of the Delphi more than 75% of panellists assigned a score of 4 or more reaching agreement (25th percentile ≥ 4). These statements also reached a high consensus (IQR < 2) as the panellists assigned similar scores. The 8 statements are:

8. *When treating a depressed patient who has failed to respond to two antidepressants, I do tend to add lithium first.*

For this statement, the IQR is 1.5 in the second round with a median value of agreement of 5% and 52% of respondents changed their answer between the first and second round. Only two cases assigned a score lower than 4 with the following motivations and one a score higher than 5.5:

- I prefer augmentation with atypical antipsychotics rather than stabilizers: score 3
- There are other possibilities as re-evaluation of possible organic causes, combination of antidepressant drugs, augmentation with atypical antipsychotics, esketamine: score 3
- Based on my clinical experience, I obtained the most effective response using drugs: score 7.

10. *When treating a depressed patient who has failed to respond to two antidepressants, I believe the best option is to combine esketamine (if currently available or when available in my centre).*

For this statement the median value is 6, the IQR is 2 in both rounds and 27% of respondents changed their answer between the first and second round. Only one psychiatrist answered out of range with this motivation:

        •There are more accessible and cheaper enhancement strategies, as well as indicated, given the prevalence of bipolar depression: score 4.

11. *I am satisfied with the efficacy of lithium and/or antipsychotics as augmentation therapies for patients with TRD.*

For this statement, half of the panellists changed their minds between first and second round but at the end 100% of them agreed with the statement and assigned a score between 4 and 5. The IQR is 1 in both rounds. Three psychiatrists assigned a higher score with respect to the median declaring:

- Based on my clinical experience, I believe that augmentation is very useful: score 6
- I hardly do not get satisfactory results: score 7.

13. *After obtaining a satisfactory response to treatment in a patient with TRD, long-term maintenance of therapy is essential.*

For this statement at the second round, the 77% assigned score 7 (maximum agreement) and the rest of the group assigned score 6. Only 20% of panellists changed their answer, the lowest value of the entire survey, the IQR at the second round is 0, indication of the total agreement and consensus.

14. *In my opinion, most patients with TRD can be treated with esketamine nasal spray in a community outpatient setting, without difficulties.*

The median value of the agreement is 6, with values clearly in disagreement with the statement even at the second round. The IQR is 2 even at the second round, with 29% of panellists that varied from first and second round. It is of maximum interest to describe the motivations of the psychiatrists in disagreement with the statement at the second round:

- Based on my experience, I believe that at least the induction phase should be administered in a hospital environment, possibly Day Hospital (DH), mainly due to the risk associated with dissociative phenomena: rate 2
- The cost of the drug, the consequent difficulties in the governance of pharmaceutical expenditure and the need for the presence of personnel in the autonomous area make it difficult to think that it will be easy: rate 1
- I agree but often the drug is not available: rate 4.

15. *Educational support for patients helps to make the best use of the therapeutic opportunity offered by esketamine nasal spray.*

At the second round, 100% of the participants reached agreement (score 6–7) and consensus, and the IQR is 1. The 27% varied their answers from first and second round.

16. *In my daily reality, I have adequate and sufficient resources (staff, logistics, facilities, etc.) to provide patients with TRD with the best possible care.*

The median value of agreement is 5. The IQR for this statement is wide: 3 in the first round, 2 in the second one; the 39% of respondents varied the answers. There are motivations in disagreement with the statement even at the second round:

- The cost of the drug, the consequent difficulties in the governance of pharmaceutical expenditure and the need for the presence of personnel in the autonomous area make it difficult to think that it will be easy: rate 2
- Unfortunately, the lack of doctors and inadequate facilities make it difficult to give adequate answers: rate 2.

17. *In my opinion there are aspects of professional responsibility that the clinician must take into consideration in order to prefer, when possible, drugs with approved clinical indications for patients with TRD.*

Almost all the participants assigned a score between 6 and 7 (maximum agreement); the IQR is 1 for both rounds. 32% of the participants varied their answers.

There are four statements classified as high consensus, but with indecision in the agreement (25th percentile < 4 and 75th percentile > 4 and IQR ≤ 2).

From a practical point of view, this means that the panellists assigned similar scores, but these scores are in the middle of the Likert scale showing a moderate agreement with the statement: less than 75% but more than 50% (in this case) assigned a score ≥ 4.

1. *In my opinion, environmental and personological factors are among the main factors responsible for the non-response to treatment of almost all patients with TRD.*

48% of the respondents varied their answers between first and second rounds.

4. *In my opinion, a shared strategy is being pursued in Italy (based on guidelines, evidence-based treatments, diagnostic-therapeutic pathways) for the management of patients with TRD.*

52% of the respondents varied their answers between first and second rounds.

5. *Based on my clinical experience, I believe that after the second treatment failure there is a clear reduction in remission chances.*

61% of the respondents varied their answers between first and second round (the widest variation recorded among the survey results). One member of the panel in the second round assigned a score out of the range that was reached in the first round, closely in agreement with the statement:

- As reported in the literature, after the first two failures, the possibility of an improvement decreases drastically: score 7

For the following statement the panel reached the consensus with an IQR value of 2, but the mean value of 4.5 (3–5 at the second round) classified it as indecision. In particular, 36% of voters assigned a score of 3 and 47% voted 5 or 6.

9. *In treating a depressed patient who has failed to respond to two antidepressants I do tend to associate psychotherapy first.*

38% of the respondents varied their answers between first and second round. One psychiatry gave a motivation for his answer in disagreement with the statement, while two were more in agreement than the others:

- It is often not feasible in a territorial context: score 2.
- Because I believe that there may be personality factors that interfere with the response: score 7.
- In my opinion, psychotherapy is essential in cases of resistance to treatment as very often the resistance is associated with important psychological or environmental factors: score 7.

The last group is represented by 5 statements classified as indecision in the agreement (the 25th percentile < 4, 75th percentile > 4) and low consensus with the statement (IQR ≥ 3).

From a practical point of view, this means that there is a low consensus among the panellists with score ranging from 2 to 7 in the Likert scale.

2. *In my clinical practice, I also consider as treatment resistant a patient with incomplete improvement of symptoms (partial response) after an adequate period of treatment.*

The panel did not reach the consensus around this statement with a IQR value of 3, and the answers values varying from 2 to 6 in the first round to 3–6 in the second round. 21% of the respondents assigned a score of 2 and 32% assigned a score of 6. 38% of the respondents varied their answers between the first and the second rounds.

3. *In clinical practice, I usually use scales/questionnaires for the diagnostic classification and/or evaluation of the clinical course of the patient with depression.*

The panel did not reach the consensus around this statement with a IQR value of 3, with a huge variability in the scores of the answers at the second round (from 2 to 7). 30% of the respondents assigned a score of 3 and the 39% assigned a score of 6. 50% of the respondents varied their answers between the first and the second rounds.

Those who answered out of the range of the first round are the following:

- I do not use them in current practice, mainly seeing patients in the private practice: score 2.
- It is often not feasible, given time constraints. The electronic record systems provided by the public service are not adequate for the integration of the psychometric evaluation: score 2.
- Fully agreement because I work in a university and research setting: score 7.

6. *When I treat a depressed patient, if after 3–4 weeks there is no response, I decide to change the antidepressant therapy.*

The panel did not reach the consensus around this statement with a IQR value of 3 in both rounds, with a mean value at the second round of 4.5 (indecision). 55% of the respondents varied their answers between the first and the second round.

At the second round 1 psychiatrist gave a motivation completely in disagreement with the statement, while on the other hand 2 colleagues gave motivations fully in agreement:

- Considering the latency of response to oral serotonergic drugs (at least 3–4 weeks) before changing the dosage or the antidepressant or boosting with lithium or other drugs, I usually wait 6–7 weeks from the start of treatment: score 1.
- I believe that it is correct to change therapy because, according to my experience, it is unlikely that after this period if there has been no clinical improvement, this will occur later: score 7.
- I believe that it is essential to evaluate early signs of response, including side effects, as predictors of an effective response at 2 months: score 7.

7. *When treating a depressed patient who has failed to respond to two antidepressants, I believe that the best option is to combine an antipsychotic, such as quetiapine.*

The panel did not reach the consensus around this statement with a IQR value of 3 in both rounds, with a mean value at the second round of 3, with the first and third quartile of 3 and 5 respectively the level of agreement is classified as indecision. At the second round the answers are spread on all 7 possible scores, about 20% for each of the possible scores between 2 and 5. 43% of the respondents varied their answers between the first and second rounds. Two motivations strongly in disagreement and three closely in agreement with the statement are recorded:

- I prefer to use aripiprazole: score 1.
- I rarely choose quetiapine for augmentation in these cases: score 1.
- Trying an antipsychotic is a valid augmentation strategy: score 6.
- It depends on the specific psychopathological dimensions of the patient: score 6.
- Several evidence have shown that it is not advantageous in terms of efficacy to further switch antidepressants, but rather to use augmentation or combination strategies: score 7.

12. *I use (or I will use) esketamine nasal spray only after non-response to the available augmentation/increase strategies.*

The panel did not reach the consensus around this statement with a IQR value of 2.5 at the second round, with a mean value of 5, with the first and third quartiles of 3 and 5.5, respectively; the level of agreement is classified as indecision. 48% of the respondents varied their answers between the first and the second rounds.

## Discussion

A high level of consensus and agreement was achieved for 8 out of the 17 statements (Table 2).

**Table 2:** Number and text of the 8 statements with high consensus and agreement

Number	Statement
8.	When treating a depressed patient who has failed to respond to two antidepressants, I do tend to add lithium first
10.	When treating a depressed patient who has failed to respond to two antidepressants, I believe the best option is to combine esketamine (if currently available or when available in my centre)
11.	I am satisfied with the efficacy of lithium and/or antipsychotics as augmentation therapies for patients with TRD
13.	After obtaining a satisfactory response to treatment in a patient with TRD, long-term maintenance of therapy is essential
14.	In my opinion most patients with TRD can be treated with esketamine nasal spray in a community outpatient setting, without difficulties
15.	Educational support for patients helps to make the best use of the therapeutic opportunity offered by esketamine nasal spray
16.	In my daily reality, I have adequate and sufficient resources (staff, logistics, facilities, etc.) to provide patients with TRD with the best possible care
17.	In my opinion there are aspects of professional responsibility that the clinician must take into consideration in order to prefer, when possible, drugs with approved clinical indications for patients with TRD

Combining the results of different statements, it can be inferred the importance of lithium and antipsychotics in managing TRD, but also that incorporating esketamine nasal spray as an augmentation therapy is the most suitable approach for treating TRD patients. This highlights the importance of prioritizing drugs with approved clinical indications for TRD patients, whenever possible.

Moreover, there is consensus and agreement about the fact that the majority of TRD patients can receive treatment with esketamine nasal spray in a community outpatient setting without significant difficulties. There is acknowledge about the benefits of providing educational support to patients in maximizing the therapeutic benefits of esketamine nasal spray.

The medical community emphasizes the significance of maintaining long-term therapy after achieving a satisfactory treatment response in TRD patients, agreeing that there are adequate and sufficient resources, including staff, logistics, and facilities, to provide the best possible care to TRD patients.

However, when considering the statements related to the "Clinical characteristics and diagnosis of patients with TRD" (statements 1, 2, 3), there was no agreement regarding the role of environmental and personological factors in non-response to treatment. The lack of consensus on partial response (statement 2) may reflect international debate and uncertainties, also at the level of scientific societies, surrounding the definition of TRD. Additionally, there was a lack of strong consensus regarding the use of scales/questionnaires, which may be due to the perceived burden of using them in busy clinical practice, with a shortage of personnel, despite their ability to improve diagnosis and monitoring of the disease over time.

Regarding the "Treatment journey and its organizational implications" (statements 4, 5, 6, 13, 16, 17), the surveyed physicians agreed on the need for long-term treatment and providing TRD patients with appropriate and approved treatments. However, there was low consensus and indecision about waiting 3–4 weeks before modifying an ineffective antidepressant treatment (statement 6). In daily practice, the discrimination between a partial response and a lack of response may be blurred. Different approaches have been proposed over the years regarding the duration of an antidepressant treatment cycle before modification. In routine clinical practice, an inappropriate delay in treatment change is often observed, as documented in a TRD cohort study [20].

The importance of a common treatment strategy pathway (statement 4) was recognized by most participants. Surprisingly, although there was consensus on statement 5, which stated that there is a clear reduction in remission chances after the second treatment failure, there was indecision regarding the agreement. This is contrary to the literature [1] probably because in Italy, traditionally, direct clinical experience is crucial with an equal significant role compared to guidelines and evidence-based treatments.

Statement 13 obtained wide consensus and agreement as all panellists understood the importance of long-term maintenance therapy after achieving a satisfactory treatment response in TRD patients. Treatment continuation is a collaborative decision made by the clinician and the patient. Reminder calls, text messages, or digital calendars can encourage adherence in the absence of treatments with documented long-term effects even after withdrawal [4].

From an organizational perspective, the surveyed Italian healthcare professionals expressed moderate consensus that they have adequate and sufficient resources (staff, logistics, facilities, etc.) in their daily practice to provide the best possible care to TRD patients (statement 16). Despite discussions about the underfunding of the mental health system, clinicians strive to ensure that patients receive the most appropriate treatments.

The statements focused on "Antidepressant treatment in routine clinical practice" (statements 7, 8, 9, 10, 11, 12, 14, 15) highlighted the absence of approved drugs for TRD treatment before the arrival of esketamine nasal spray. However, there are various pharmacological and non-pharmacological approaches commonly used. The results indicated greater confidence in augmentation with lithium compared to quetiapine, and a moderate level of satisfaction with this strategy.

In line with clinical practice, there was indecision regarding the association of psychotherapy in TRD patients (statement 9). This is consistent with an Italian Real-World Study where psychosocial therapy was prescribed to only 7% of TRD patients [5], likely due to limited availability and use of such techniques in Italian public mental health departments. The literature suggests that a combination of pharmacological and psychological approaches may be the most effective treatment for most people with TRD in terms of acute response and relapse prevention [21].

There was consensus and agreement that TRD can be treated with esketamine nasal spray after two antidepressant failures (statement 10), and it can be administered in a community outpatient setting without difficulties (statement 14). This is consistent with literature reporting the safety and tolerability of esketamine nasal spray in real-world settings [22] and suggests that well-trained nurses can manage TRD patients following esketamine administration and monitor adverse events [10].

The importance of educational support for patients to make the best use of the therapeutic opportunity offered by esketamine nasal spray (statement 15) aligns with literature findings since poor understanding of the therapy area can lead to patient non-compliance [4, 22].

Finally, regarding the use of esketamine nasal spray, there was low consensus and indecision about using esketamine only after non-response to available augmentation/increase strategies (statement 12). This could be attributed to the recent approval of the drug and the need for better education among healthcare professionals regarding its place in therapy. There is a lively debate within the medical community about the treatment of TRD for several reasons: from the absence of consensus about TRD definition up to the need of systematic reviews [23]. As far as today, currently available augmentation strategies are often off-label or based on weak evidence (limited samples). TRD patients represent an under-researched clinical population (most clinical trials of investigational agents in MDD exclude patients with TRD) with relevant morbidity and mortality. This is the reason why novel interventions that offer meaningful benefit are so eagerly awaited and welcomed [15].

As for other Delphi studies the limitation of the results can be attributed to the methodology itself, based on opinions; therefore, the study cannot be considered as substitute for forms of evidence high above the pyramid.

## Conclusions

This Delphi Panel showed that there is a wide heterogeneity in the management of TRD patients, and a compelling need of standardised strategies and treatments specifically approved for TRD. A high level of consensus and agreement was obtained about the importance of adding lithium and/or antipsychotics as augmentation therapies for patients with TRD, but also about the identification of esketamine nasal spray as the best option when treating a depressed patient who has failed to respond to two antidepressants. The medical community participating to the Delphi reached a high level of consensus and agreement about the possibility to treat most patients with TRD with esketamine nasal spray in a community outpatient setting, without difficulties and about the benefit of educational support for patients that are offered esketamine nasal spray. Similarly, the need for long-term maintenance therapy and the availability of adequate and sufficient resources (staff, logistics, facilities, etc.) to provide patients with TRD with the best possible care obtained a high level both of consensus and agreement. In conclusion, is remarkable the high consensus and agreement in the medical community about the opportunity to prefer drugs with approved clinical indications for patients with TRD.

### Supplementary Information

**Additional file 1.** Median and 25th percentile, 75th percentile and IQR for each statement and round.

## Data Availability

The data sets used and/or analysed during the current study are available from the corresponding author on reasonable request.

## Abbreviations

DH: Day Hospital
EMA: European Medicines Agency
HCP: Health Care Professionals
IQR: Interquartile range
MAOIs: Monoamine oxidase inhibitors
MDD: Major depressive disorder
NHS: National Health System
NMDA: Ionotropic *N*-Methyl-d-Aspartate
NS: Nasal spray
SGA: Second-generation antipsychotic
SmPC: Summary of product characteristics
SNRI: Serotonin-norepinephrine reuptake inhibitors
SSRI: Serotonin reuptake inhibitors
STAR-D: Sequenced treatment alternatives to relieve depression
TCA: Tricyclic antidepressants
TRD: Treatment-resistant depression
XR: Extended release
