# Supplementary material for: Nationwide consensus on the clinical management of treatment-resistant depression in Italy: a Delphi panel
Source: Ann Gen Psychiatry. 2023 Nov 23;22:48. doi: 10.1186/s12991-023-00478-7 (PMC10668442; doi:10.1186/s12991-023-00478-7)

**Title**: TRD - Delphi	**Version:** 1.0<br>**Date:** Feb, 03 2023

**Author**	
**Name**	**Title**
Valentina Panetta	**Senior Biostatistician**

Methodology

During the first meeting, the board identified 17 statements that were submitted via web-based administration to the expert panel.

This method allowed each expert to answer without interacting with the others and maintaining anonymity. Each question could be answered according to level of agreement/disagreement on a 7-point scale (1 no agreement, 7 maximum agreement).

At the end of first round, statistician calculated 25° and 75° percentile (Interquartile range) of each statement.

In the second round the experts were invited to converge their answer within the interquartile range (IQR) or to provide reasons in case their evaluation is outside the range.

At the end of second round median and IQR were calculated for each statement and at the board's meeting results and reason were evaluated.

Statements were classified considering 25° percentile, 75° percentile and IQR.

Consensus was defined as IQR <=2 and agreement with the statement when the 1st quartile was >=4:

Agreement and consensus: 25° percentile ≥4 and IQR ≤2 and IQR different from 4-4.
Agreement and low consensus: 25° percentile ≥4 and IQR ≥3.
Indecision and consensus: 25° percentile ≥4 and IQR 4-4 OR 25° percentile <4 and75° percentile >4 and IQR ≤2.
Indecision and low consensus: 25° percentile <4, 75° percentile >4 and IQR ≥3.
Disagreement and consensus: 25° percentile <4, 75° percentile ≤4 and IQR ≤2.
Disagreement and low consensus: 25° percentile <4, 75° percentile ≤4 and IQR ≥3.

Statement 1

	First Round	Second Round
Number	60	56
Median	4	4
25° Percentile	3	3
75° Percentile	5	5
Interquartile range	2	2

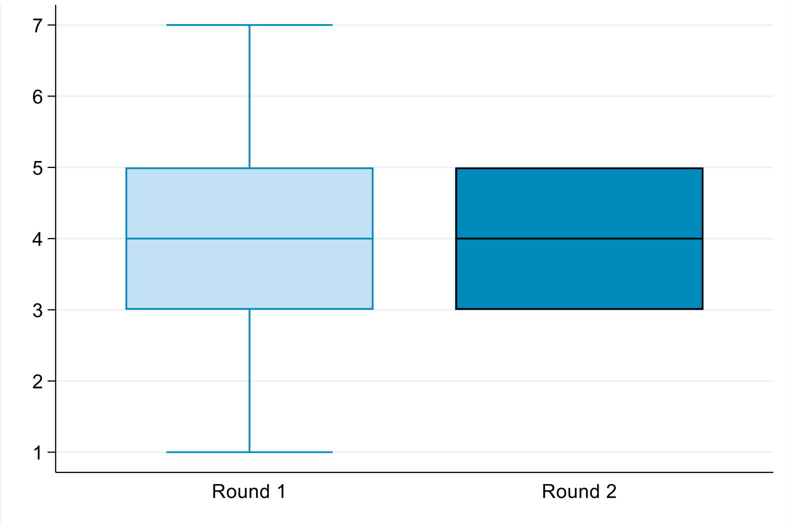

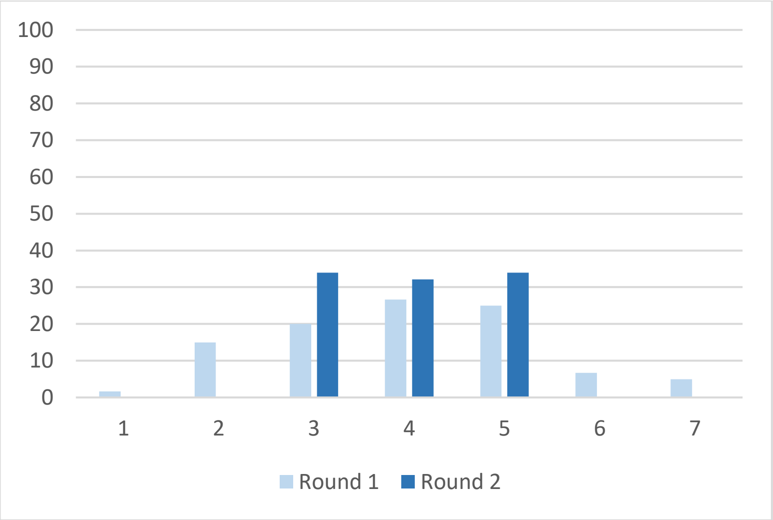

Statement 2

	First Round	Second Round
Number	60	56
Median	5	5
25° Percentile	2	3
75° Percentile	6	6
Interquartile range	4	3

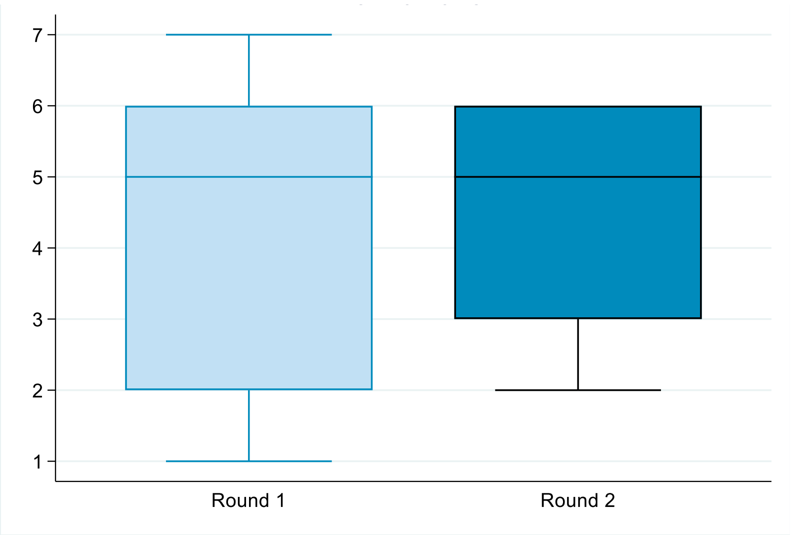

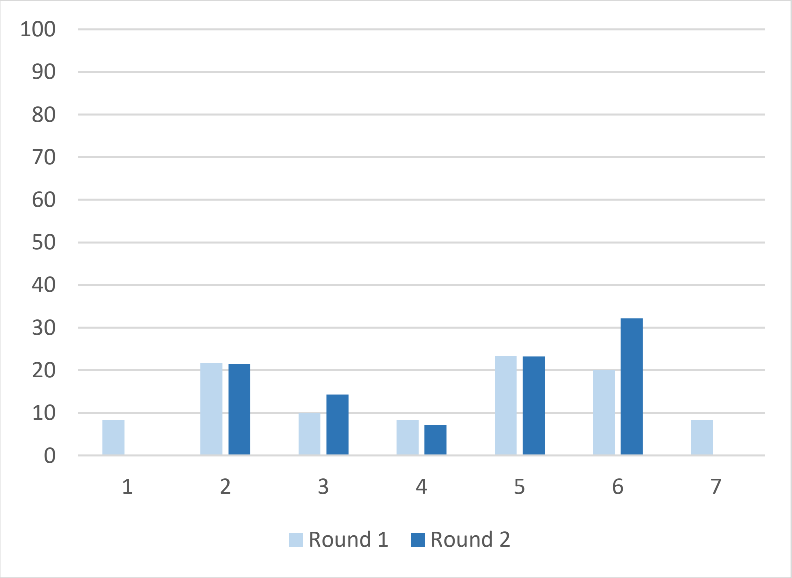

Statement 3

	First Round	Second Round
Number	60	56
Median	5	5
25° Percentile	3	3
75° Percentile	6	6
Interquartile range	3	3

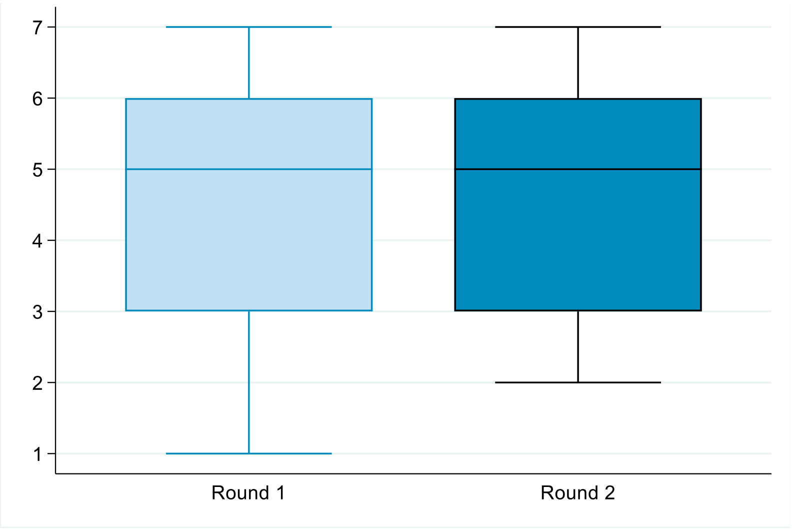

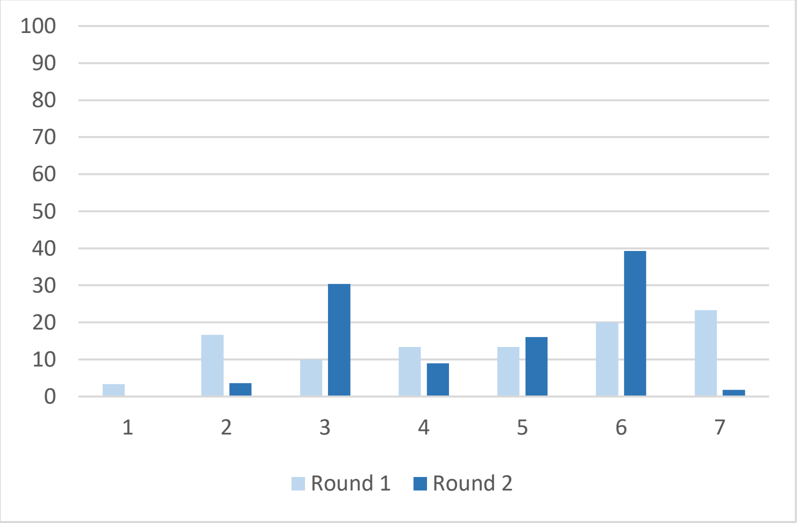

Statement 4

	First Round	Second Round
Number	60	56
Median	3	4
25° Percentile	2	3
75° Percentile	5	5
Interquartile range	3	2

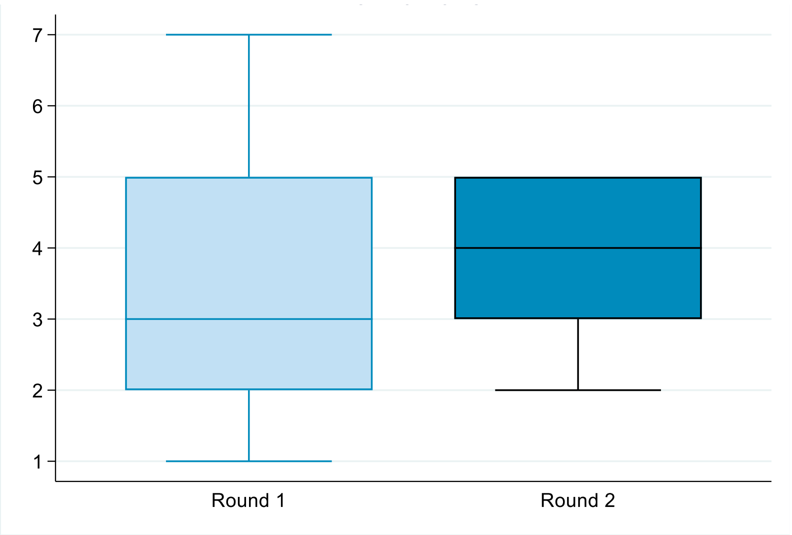

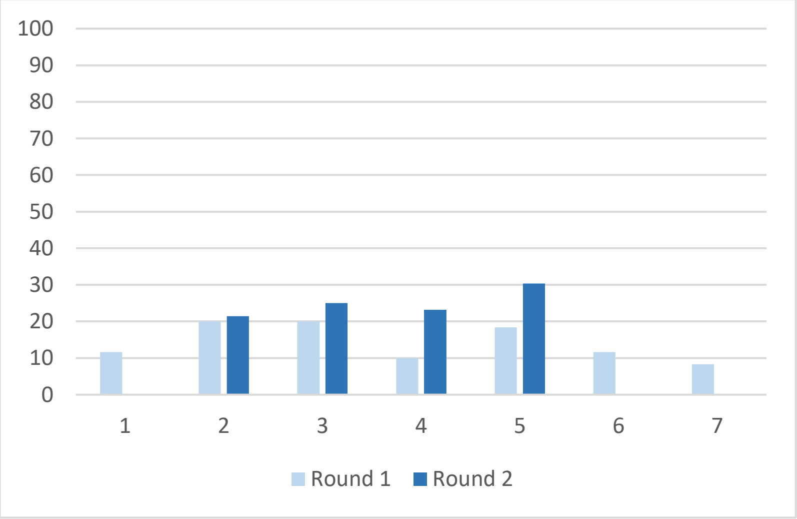

Statement 5

	First Round	Second Round
Number	60	56
Median	4	4
25° Percentile	3	3
75° Percentile	5	5
Interquartile range	2	2

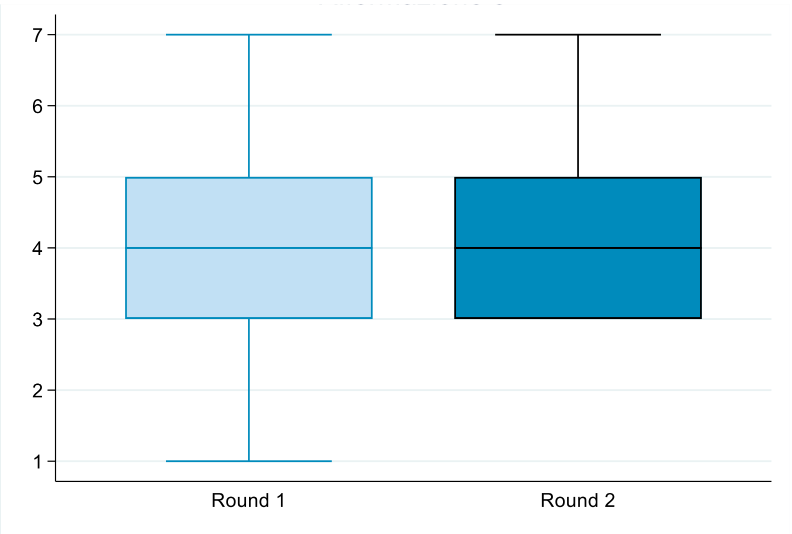

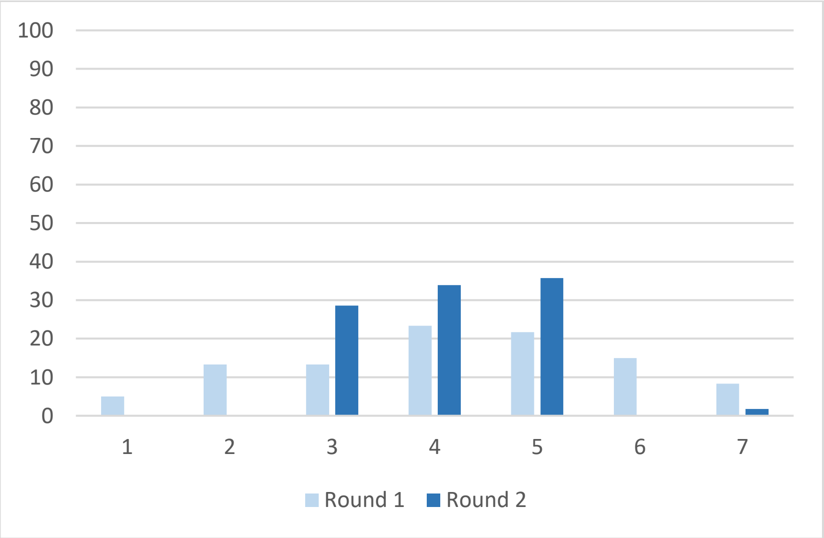

Statement 6

	First Round	Second Round
Number	60	56
Median	4	4.5
25° Percentile	3	3
75° Percentile	6	6
Interquartile range	3	3

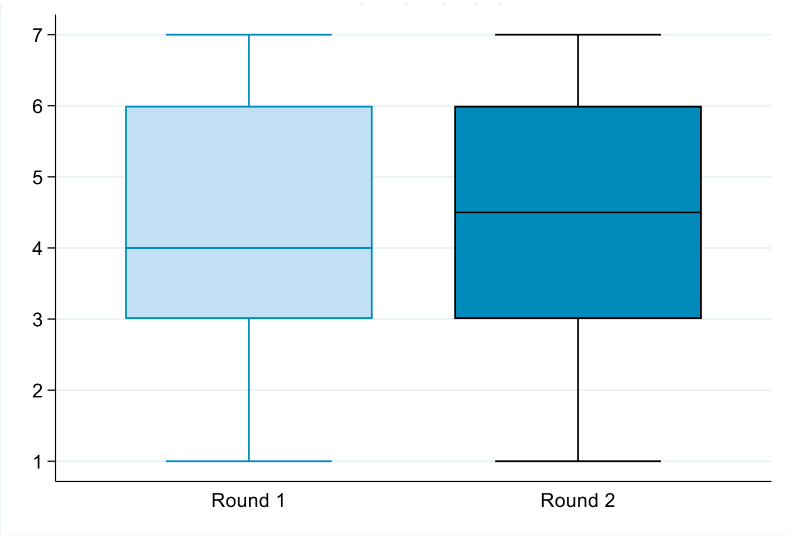

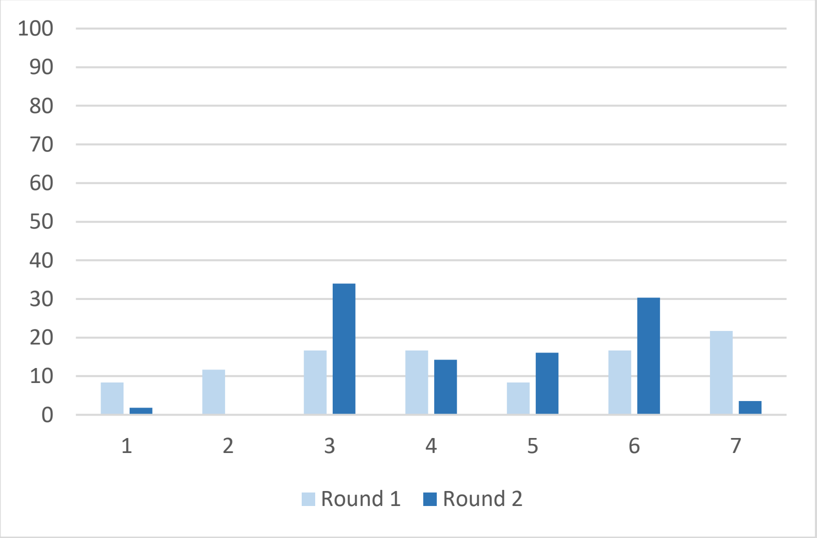

Statement 7

	First Round	Second Round
Number	60	56
Median	4	3
25° Percentile	2	2
75° Percentile	5	5
Interquartile range	3	3

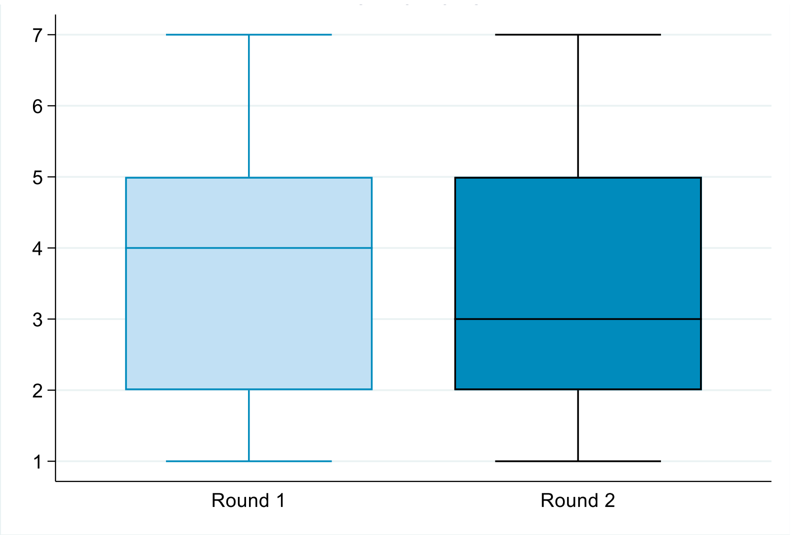

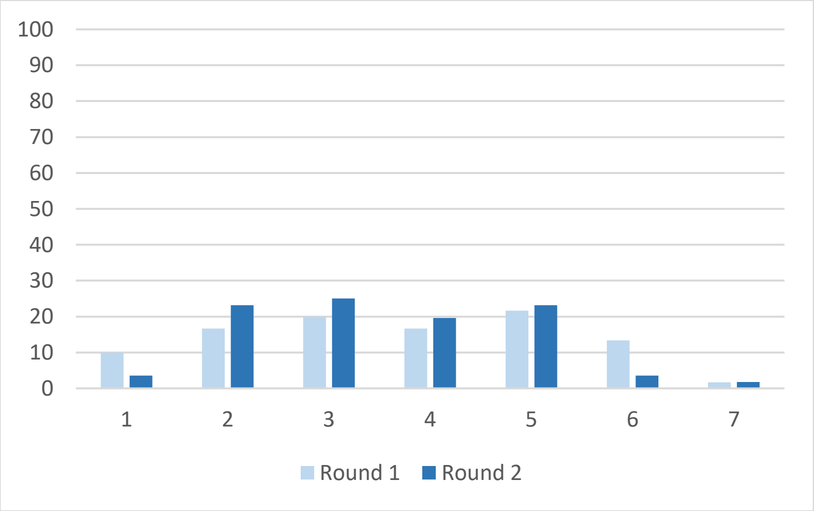

Statement 8

	First Round	Second Round
Number	60	56
Median	5	5
25° Percentile	4	4
75° Percentile	6	5.5
Interquartile range	2	1.5

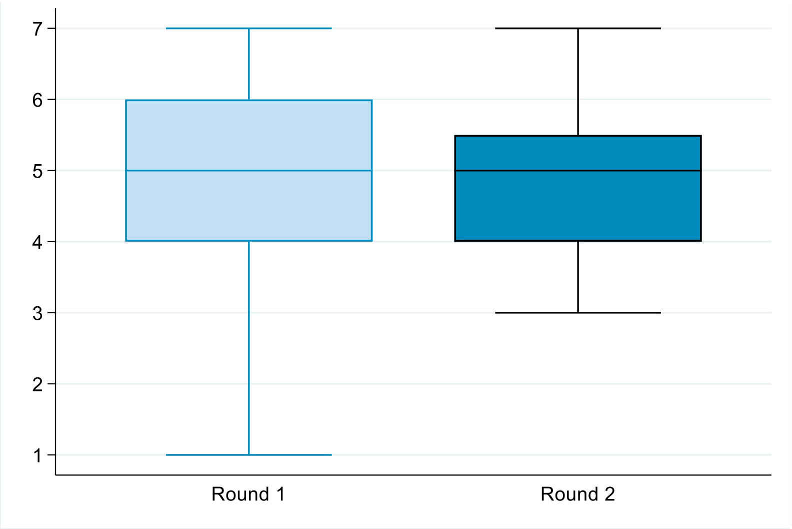

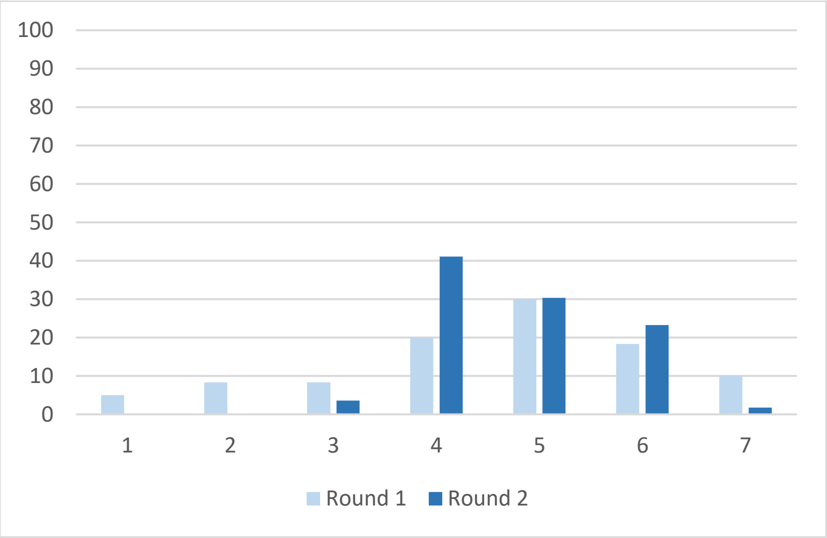

Statement 9

	First Round	Second Round
Number	60	56
Median	5	4.5
25° Percentile	3	3
75° Percentile	6	5
Interquartile range	3	2

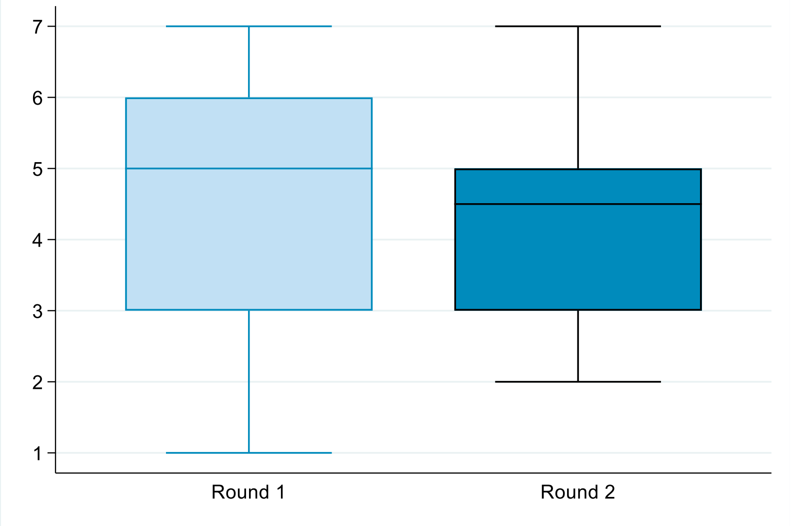

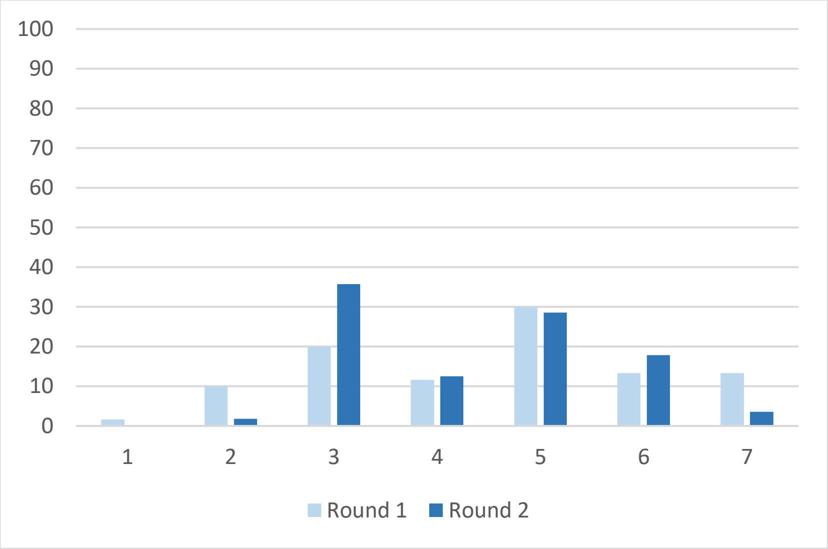

Statement 10

	First Round	Second Round
Number	60	56
Median	6	6
25° Percentile	5	5
75° Percentile	7	7
Interquartile range	2	2

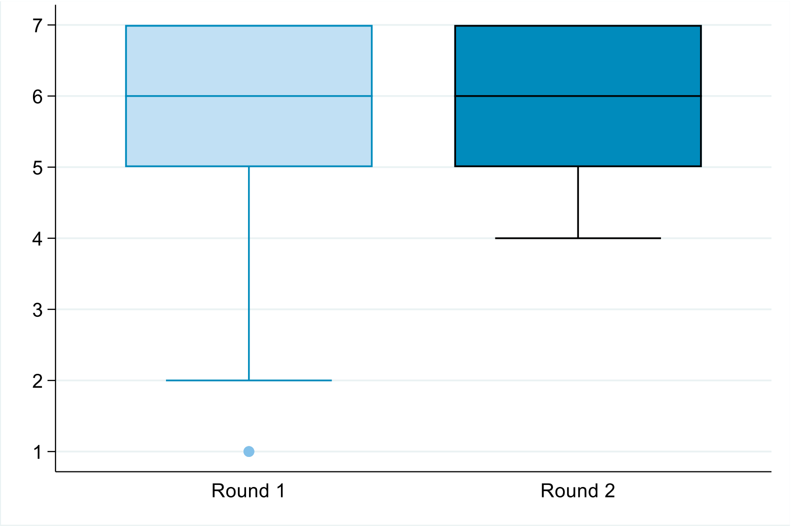

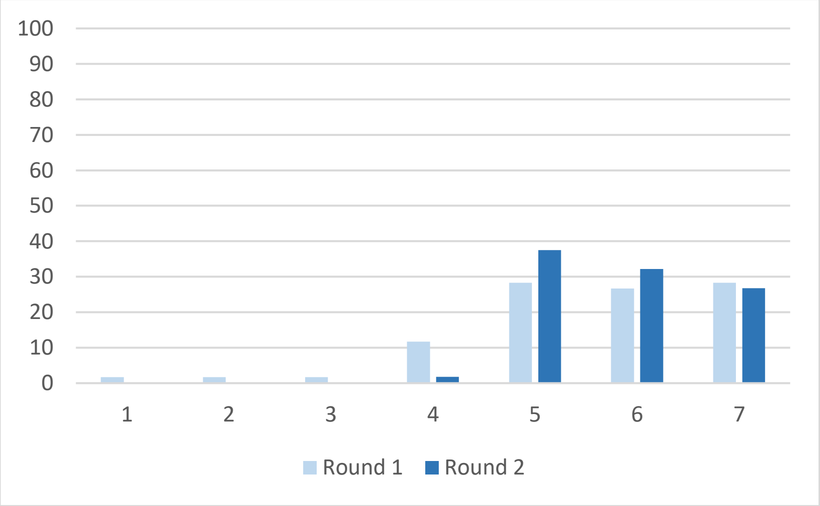

Statement 11

	First Round	Second Round
Number	60	56
Median	5	4.5
25° Percentile	4	4
75° Percentile	5	5
Interquartile range	1	1

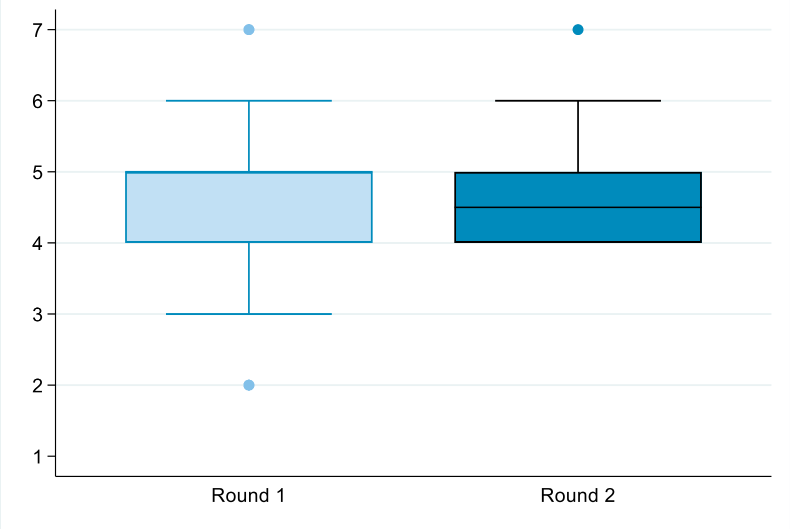

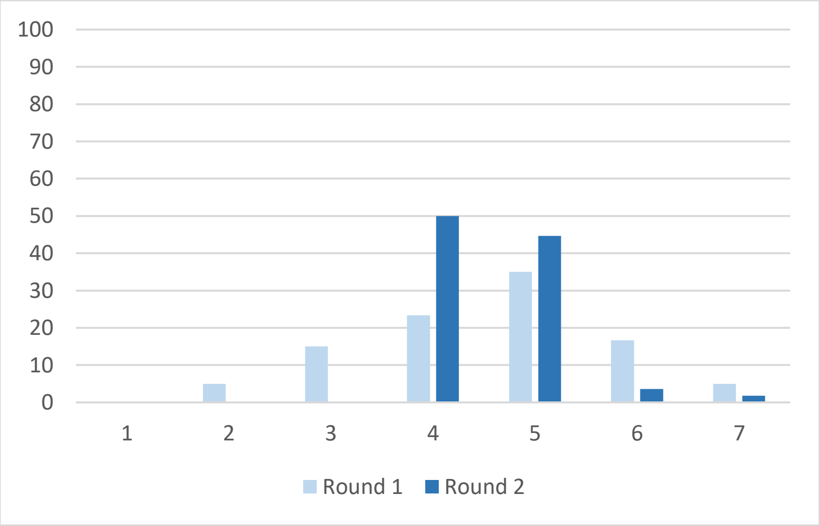

Statement 12

	First Round	Second Round
Number	60	56
Median	4	5
25° Percentile	3	3
75° Percentile	6	5.5
Interquartile range	3	2.5

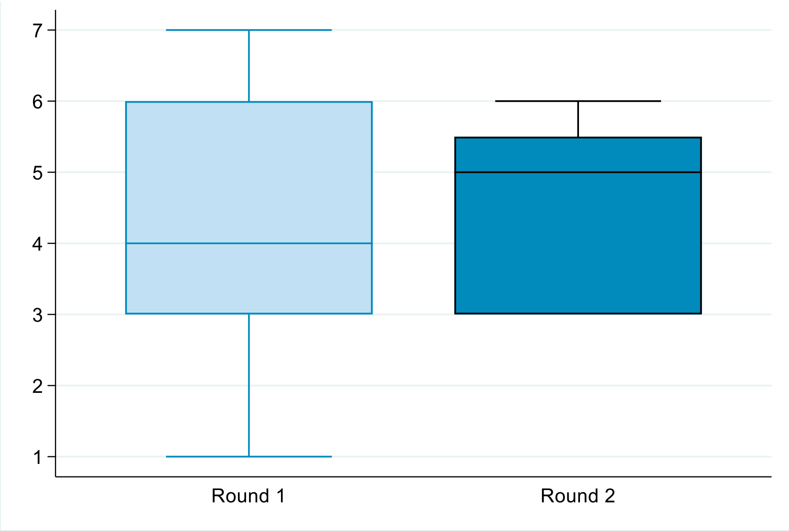

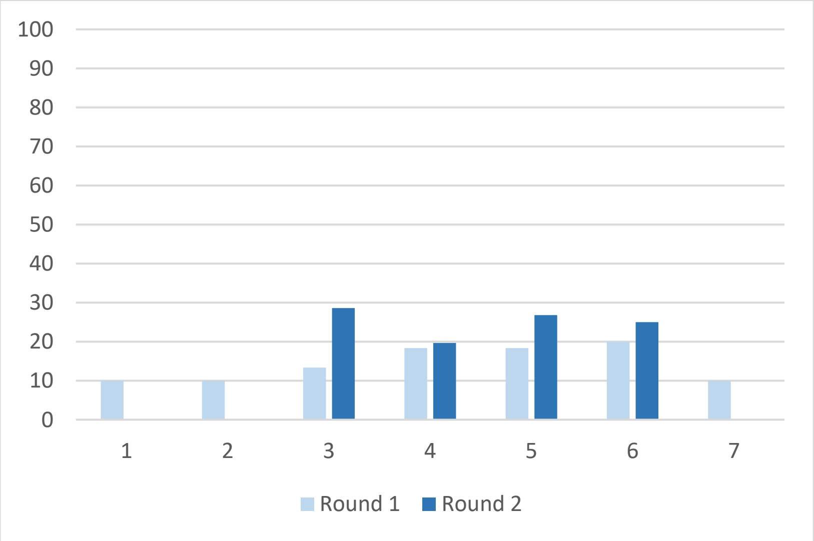

Statement 13

	First Round	Second Round
Number	60	56
Median	7	7
25° Percentile	6	7
75° Percentile	7	7
Interquartile range	1	0

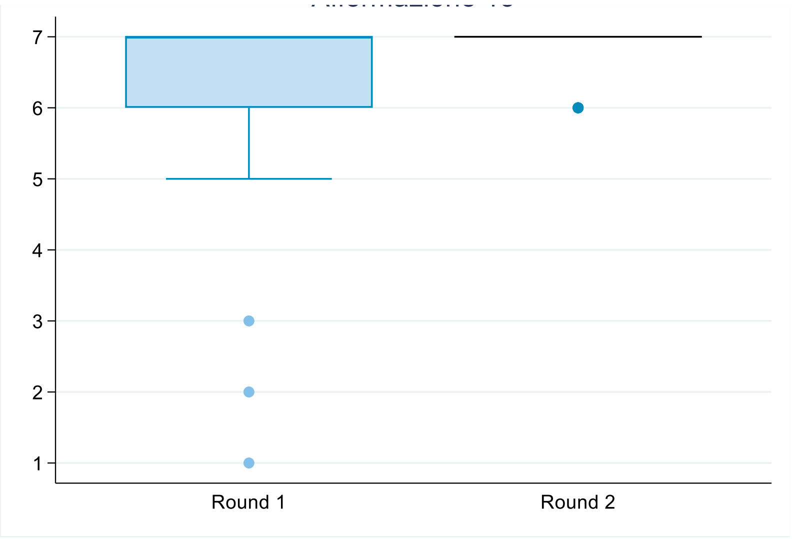

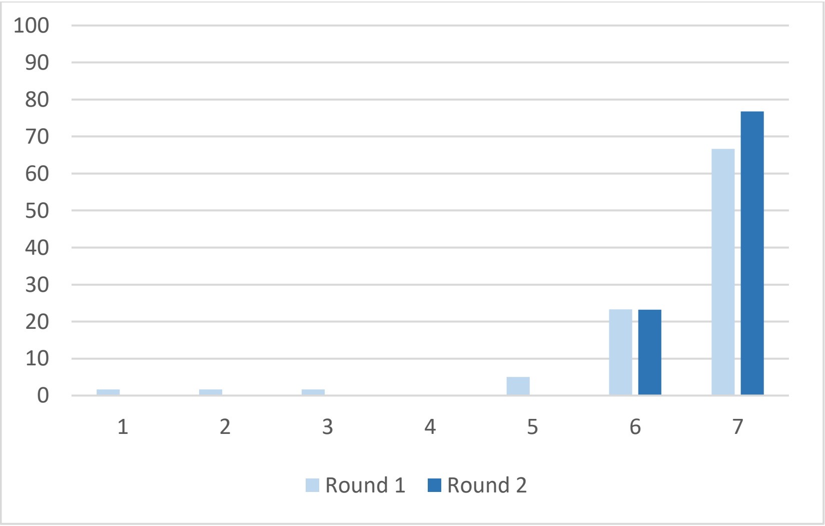

Statement 14

	First Round	Second Round
Number	60	56
Median	6	6
25° Percentile	5	5
75° Percentile	7	7
Interquartile range	2	2

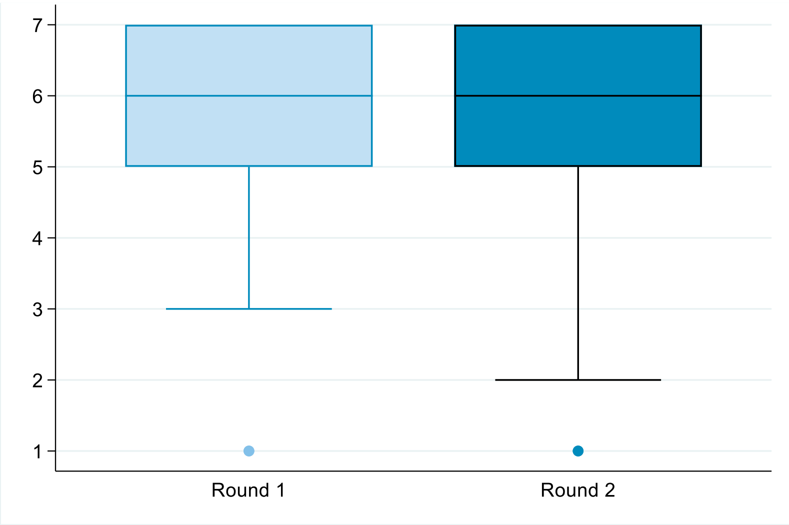

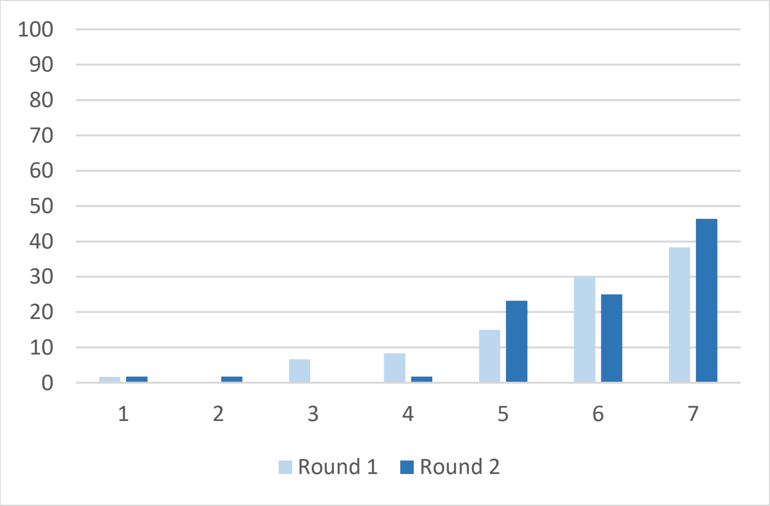

Statement 15

	First Round	Second Round
Number	60	56
Median	7	7
25° Percentile	6	6
75° Percentile	7	7
Interquartile range	1	1

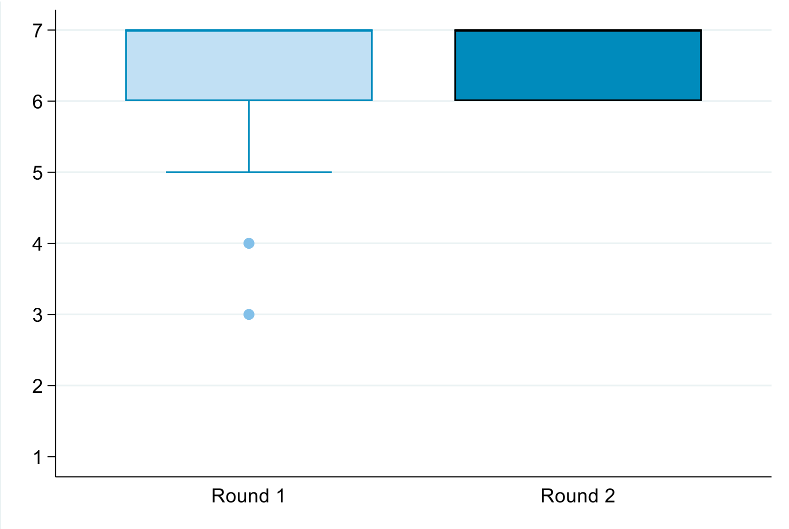

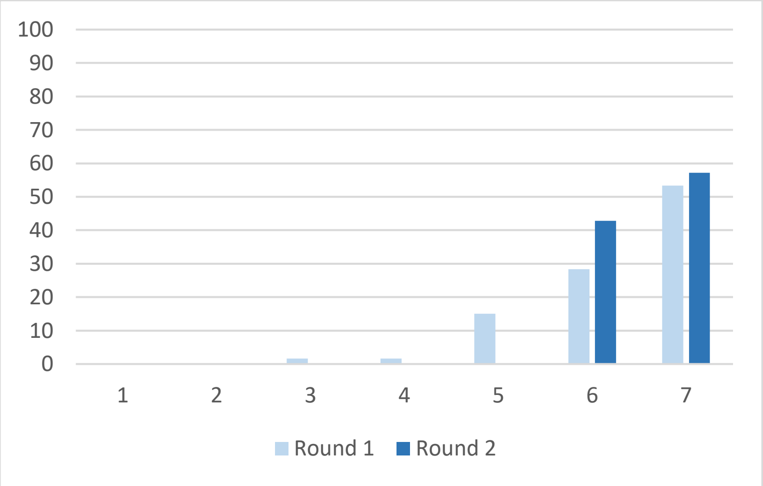

Statement 16

	First Round	Second Round
Number	60	56
Median	4.5	5
25° Percentile	3	4
75° Percentile	6	6
Interquartile range	3	2

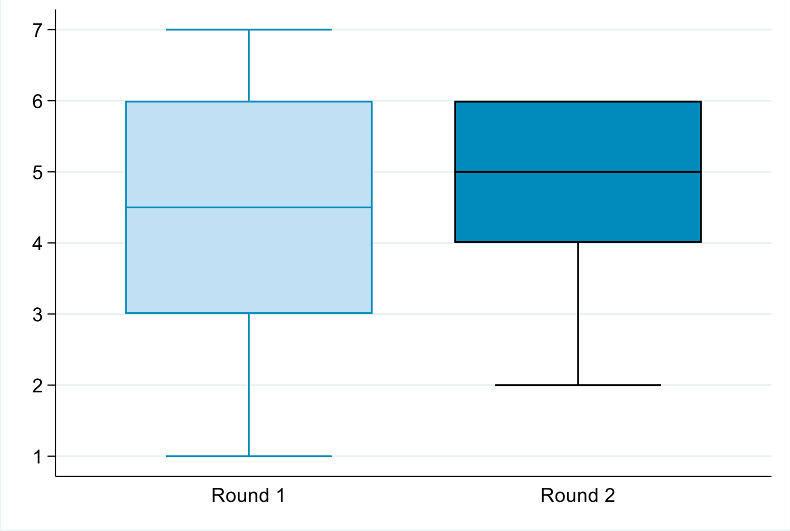

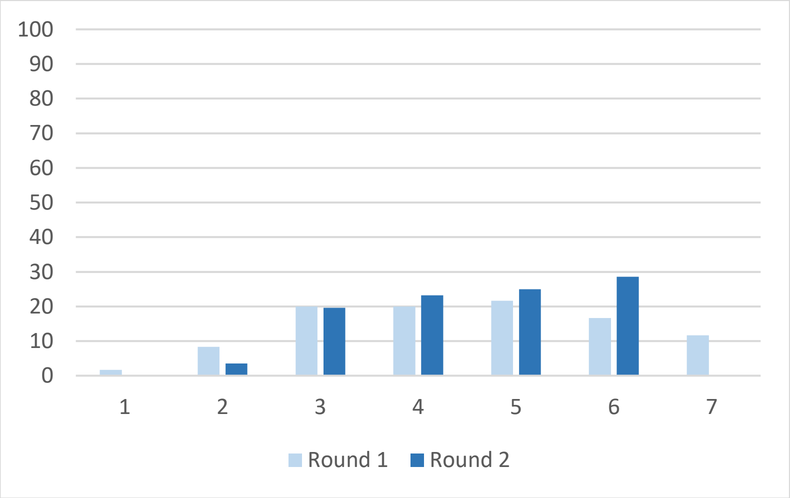

Statement 17

	First Round	Second Round
Number	60	56
Median	6	7
25° Percentile	6	6
75° Percentile	7	7
Interquartile range	1	1

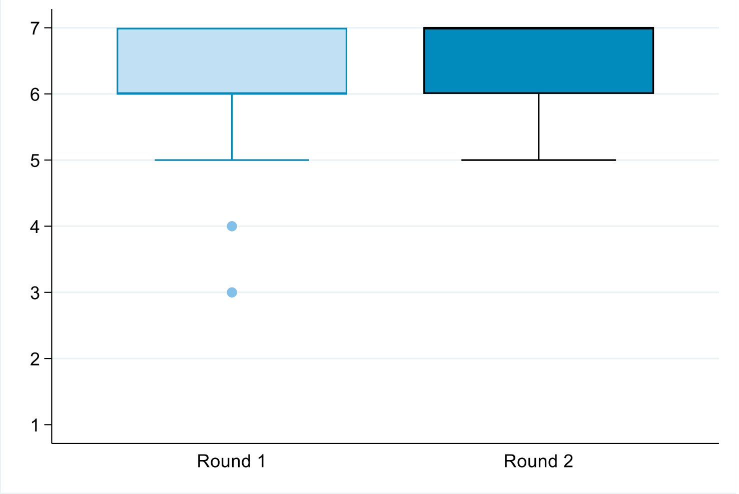

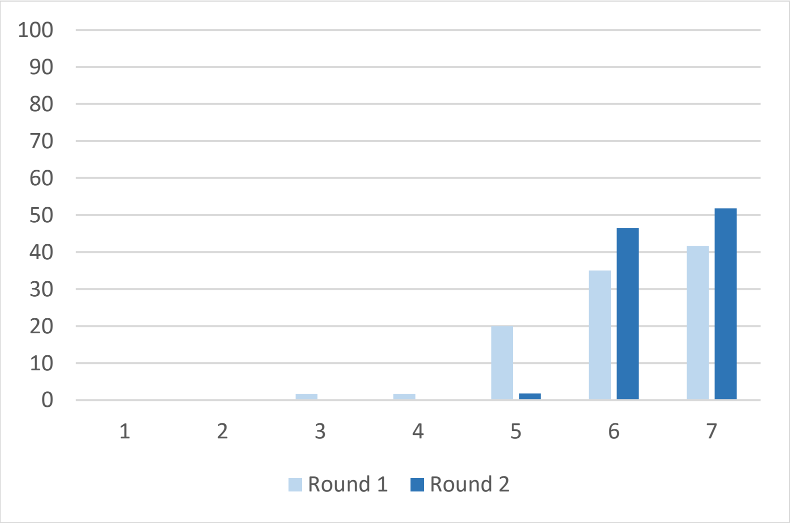

Supplement: Supplementary file 1 — Additional file 1. Median and 25th percentile, 75th percentile and IQR for each statement and round. [file 12991_2023_478_MOESM1_ESM.pdf]
